# Bedrock morphology influences rock barrens turtle nesting habitat energy dynamics

**DOI:** 10.1002/ece3.11183

**Published:** 2024-04-01

**Authors:** Brandon Van Huizen, Chantel E. Markle, Paul A. Moore, James M. Waddington

**Affiliations:** ^1^ School of Earth, Environment and Society McMaster University Hamilton Ontario Canada; ^2^ Department of Geography and Environmental Management University of Waterloo Waterloo Ontario Canada; ^3^ School of Environment, Resources and Sustainability University of Waterloo Waterloo Ontario Canada

**Keywords:** bedrock morphology, ground heat flux, heat storage, reptiles, rock barrens, species at risk

## Abstract

Energy absorption and flow through a nest is an important aspect of embryonic development in many reptile species including turtles. To date, few studies have explicitly attempted to quantify the energy flow through turtle nests, opting instead for the simplified approach offered by temperature index models. However, the quantification of the energy can provide an explicit abiotic link that can link biological models to biometeorological and ecohydrological processes and models. We investigated the energy flow through turtle nests occupying different bedrock morphologies within a Canadian Shield Rock Barren landscape, in Ontario, Canada. The taxons studied were Spotted Turtle (*Clemmys guttata*), Midland Painted Turtle (*Chrysemys picta marginata*), and Blanding's Turtle (*Emydoidea blandingii*). Nest temperature and soil moisture were measured in 2018 and 2019 using sensors placed in the soil adjacent to 12 turtle nest cavities. Three main rock morphologies were identified for each nest location, *Crevice*, *Ledge*, and *Flat* types, that are in order of decreasing bedrock percentage contact with the nest site. Ground heat flux and change in heat storage were determined using the calorimetric method for each nest, while the direction of energy flux between the atmosphere and the underlying rock was also determined. The *Crevice* nest morphology experienced the lowest ground heat flux on average (1.56 × 10^−1^ W m^−2^) and lowest cumulative heat storage (230 MJ) compared to the *Flat* (440 MJ) and *Ledge* (331 MJ) nests. However, over the diurnal cycle, large heat gains by *Flat* nests were mostly balanced out by nighttime heat losses. While *Crevice* nests saw the lowest daily heat storage gains, they experienced much lower heat losses over the evening period compared to the other nest types. Furthermore, we found that 59% of the energy is directed from the underlying bedrock into the *Crevice* nest, highlighting the importance of the bedrock in controlling thermal dynamics in the turtle nesting habitat. The lower variability in energy parameters for *Crevice* nest types can be attributed to higher amounts of nest‐to‐bedrock contact, compared to the flat nest types. Our results indicate that *Crevice* morphology may be ideal for turtles nesting at their northern limits because minimal heat loss during the evening can result in a more stable thermal incubation environment. Future conservation and habitat restoration efforts should consider the importance of bedrock morphology and prioritize the protection of *Crevice* nest sites. Furthermore, this work highlights important opportunities for potential interdisciplinary work between ecologists, climatologists, biologists, and hydrologists, specifically the integration of ecohydrological and biological models. This work also underscores the potential uncertainty of climate change impacts on turtle egg hatching success and nest sex ratios.

## Graphical abstract

Bedrock morphology can moderate turtle nest heat fluxes.

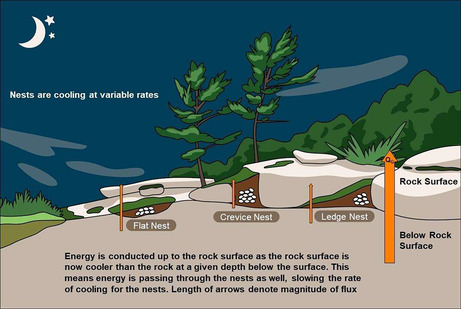

## 1 INTRODUCTION

Organisms require specific environmental conditions to survive and successfully reproduce. While the mechanisms which drive selection of environmental conditions or habitats are often species dependent, many species require nesting habitat with low variability in environmental extremes (Packard et al., 1987; Smith et al., 1992; Valenzuela et al., 2019; White & Kinney, 1974). A species' inability to tolerate environmental extremes often means they require stable temperature and moisture conditions, especially during vulnerable life stages. For example, if live births occur, vulnerable young can begin their lives in relatively low‐risk conditions (e.g., dens for mammals) where the risk of freezing and flooding is minimized (e.g., Alt, 1984). For species that lay eggs externally, such as birds and some reptiles, nest habitat requires specific insulative conditions for proper incubation and successful hatching, where incubation conditions can even influence offspring sex in some species (e.g., reptiles; Bull, 1980).

Temperature is the most commonly recorded abiotic variable when assessing nesting habitat and the incubation environment (Gedeon et al., 2010; Gillooly et al., 2002; Maziarz et al., 2017). However, energy is another important abiotic factor that directly controls nest temperature yet is understudied within the context of nest habitat conditions, likely due to the complexity in its calculation (Kearney & Enriquez‐Urzelai, 2023). The spatial and temporal variability in moisture content, nest material porosity, thermal conductivity, and specific heat capacity all influence the magnitude of the flow of energy. Measurements of nest energy dynamics are especially important as the partitioning of energy (i.e., allocation of energy to biotic and abiotic processes such as heating, photosynthesis, evaporation, etc.) across the landscape has shifted (Stephens et al., 2012) with recent climate change (IPCC, 2023). Moreover, there is a high degree of uncertainty around the effects of these shifts in energy allocation on nest habitat as energy partitioning into the nest will depend on vegetation morphological and phenological characteristics. As the climate warms and vegetation cover changes due to biometeorological and ecohydrological shifts (Richardson et al., 2013) and wildfire (e.g., Boulanger et al., 2017), alterations to nest habitat thermal regime will likely occur (Markle, Moore, & Waddington, 2020; Markle, Wilkinson, & Waddington, 2020) causing changes in nest energy absorption. The degree to which these changes occur in variables such as thermal conductivity and specific heat capacity will also depend on nest moisture content, which is of particular importance for species that make their nests within soils.

In reptile species such as turtles, nests are often laid in soil or sandy substrates which are subject to rainfall and infiltration. To maintain a stable incubation environment, it is important that turtle nesting habitat includes soils that are well drained, so as not to become too wet resulting in drowning, but still retain enough soil moisture for embryo development (Cagle et al., 1993; Markle et al., 2021; Massey et al., 2019; Obbard & Brooks, 1987; Packard et al., 1987). The presence of soil moisture has the concomitant effect of buffering temperature changes due to its high heat capacity (4000 J g^−1^ K^−1^) (Farouki, 1981). This can impact turtle nest hatch success where temperatures may not be high enough for proper incubation conditions, resulting in low hatch success and/or impact nest sex ratios in some species (Janzen, 1994; Schwarzkopf & Brooks, 1985). High hatch success rates are also related to the maintenance of adequate soil moisture conditions for the eggs (Cagle et al., 1993; Markle et al., 2021; Packard et al., 1987), as well as lower embryonic deformities (Tracy et al., 1978). Reptiles are one of the most endangered groups of vertebrates in the world (Cox et al., 2022; Stanford et al., 2020) facing population declines and habitat loss due to climate and land use changes. Therefore, it is imperative that conservation efforts focus on thoroughly identifying and understanding habitat function for turtle nesting now so that we can understand how it may change in the future.

Given the key link between energy flow and nest site suitability, quantifying ground heat flux (*Q*_G_, W m^−2^) and heat storage (Δ*S*, MJ cm^−3^) provides a natural linkage between abiotic processes and the biotic needs of organisms. Heat flux (*Q*_G_) defines the amount of energy passing through the nest, either from the surface above via incoming short and longwave radiation, or from the deeper bedrock/ground below the nest via conduction (Oke, 1987). Heat flux dictates the primary external process influencing nest temperature, either heating from the sun or the role of the immediate surrounding habitat in heating the nest. Heat storage (Δ*S*) defines the amount of energy gained or lost as sensible heat which is realized as the rise and fall of the nest or incubation temperature. However, in environmental models, heat flux and heat storage are often quantified using homogenized soil characteristics and applied to large areas (>10 m^2^, e.g., Krogh et al., 2017) where the thermal characteristics of small discrete sites (<1 m^2^), such as at the scale of a turtle nest site, are ignored to simplify calculations. Furthermore, biological models for embryo development are often not clearly coupled to these larger‐scale environmental models (e.g., Craig et al., 2020; Pomeroy et al., 2007). Recent work has shown the potential of coupling abiotic models to biotic models (Kearney & Enriquez‐Urzelai, 2023) for understanding climate change impacts on nesting habitat, and so it is important to quantify these abiotic‐biotic linkages and processes to improve our modeling capabilities and ultimately aid in conservation efforts.

At the scale of an individual nest, a complex balance exists between the coupled transport of the soil moisture and energy. To quantify resulting thermal effects, studies have used a temperature index approach (e.g., Bolton & Brooks, 2010; Obbard & Brooks, 1987; Rollinson et al., 2018) like the calculation of growing degree days in agricultural studies (e.g., White et al., 2015), as a proxy for accumulated heat energy exposure to the embryos within the eggs. However, temperature index values, although empirically based, are nevertheless an abstraction of a physical value, which can make it difficult in biogeographical studies to understand the link between abiotic and biotic processes. This is especially true when it comes to creating fully integrated ecohydrological models that extend beyond the ground and vegetation into the animal kingdom. As models of reptile egg development become more robust (e.g., Kearney & Enriquez‐Urzelai, 2023; Mitchell et al., 2016), there will be a need for a more physically accurate representation of the processes that link the surrounding landscape's function, and how it may change under climate and land use changes, to egg development. Ultimately, these complex interactions that drive everything from egg development to the evaporation of the soil moisture in the nest are driven by energy, and so quantifying heat flux and heat storage represents a unique linkage between these different fields.

With climate change, the expected increase in temperature (IPCC, 2023) will likely alter energy inputs that will directly impact turtle nesting habitat. There will likely be nonlinear responses in energy flow, making it important to quantify further changes to the energy characteristics (e.g., heat flux, heat storage) of nests. Therefore, the objectives of this study are to: (1) quantify heat flux and heat storage in turtle nests to elucidate physical factors controlling thermal stability; and (2) determine the relative contributions of energy from the atmosphere and energy from the ground in maintaining adequate nest temperature. This analysis will allow for better representation of coupled abiotic‐biotic processes in current ecohydrological models such as RAVEN (Craig et al., 2020) or CRHM (Pomeroy et al., 2007), and to better understand how the nest environment may change in the future and support the conservation of critical habitats and landscapes.

## 2 MATERIALS AND METHODS

### 2.1 Study area

This study was carried out in a 660‐ha rock barrens landscape, approximately 10–15 km east of Georgian Bay, Lake Huron Ontario. In central Ontario, Canada, the rock barrens of the Canadian Shield represent the northern limit of many species‐at‐risk turtles, including the Spotted Turtle (*Clemmys guttata*), Snapping Turtle (*Chelydra serpentina*), Midland Painted Turtle (*Chrysemys picta marginata*), and the Blanding's Turtle (*Emydoidea blandingii*), which are considered at‐risk either provincially and/or federally. This landscape is a mosaic of rock barrens made of granitic rock outcrops, upland mixed wood forests, and a variety of wetland types including peatlands and marshes (Wester et al., 2018). The post‐glacial landscape has formed sharp transition zones making for a heterogeneous land cover with distinct micro‐climatological differences (e.g., Spence & Rouse, 2001).

**FIGURE 1:**
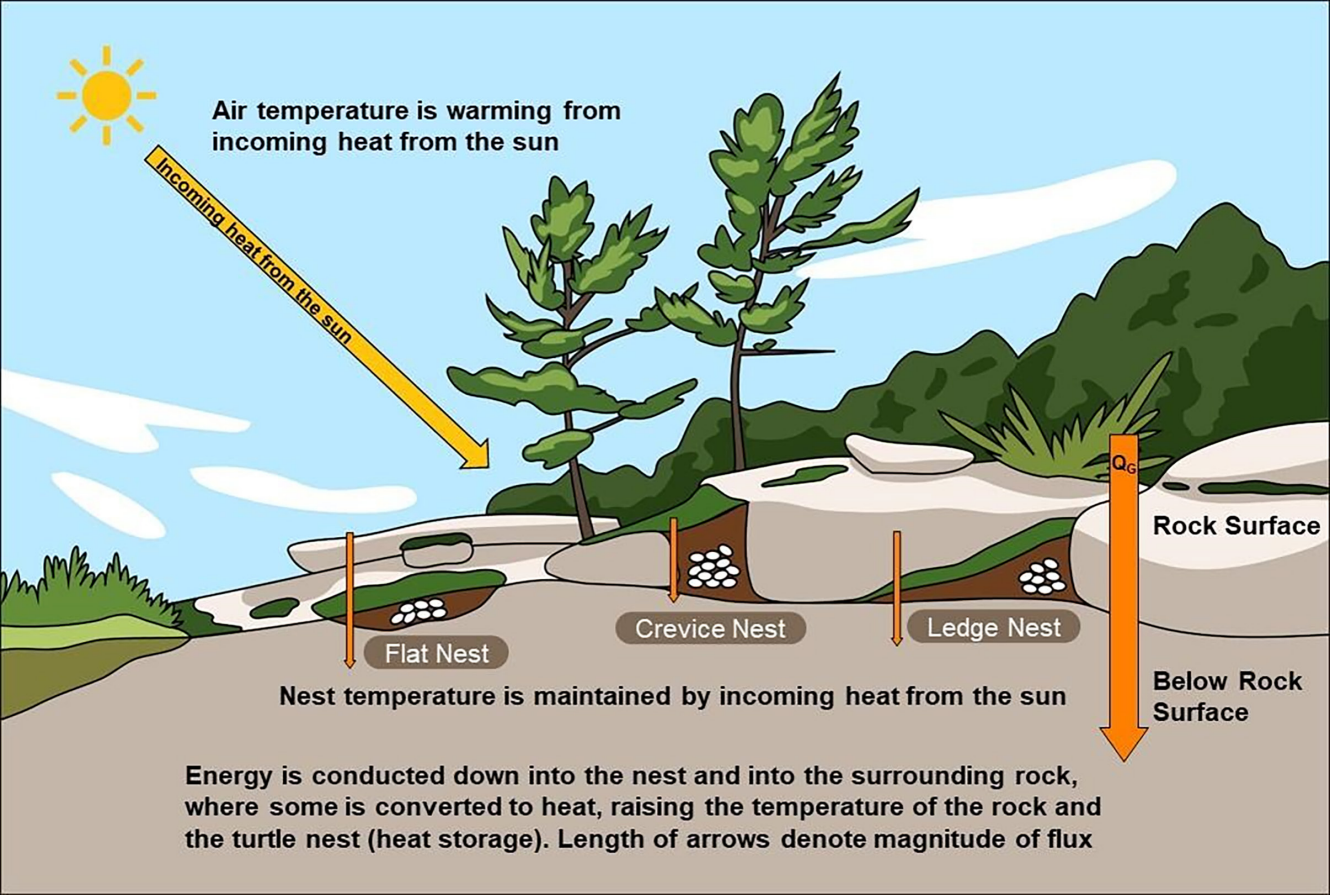
Direction of energy flow through turtle nests during the day. Energy is conducted down into the nest and into the surrounding rock, where some is converted to heat, raising the temperature of the rock and the turtle nest (heat storage). Length of arrows denotes magnitude of flux.

It is within the open rock‐barren outcrops that turtles nest within shallow soil deposits (7.5–22.2 cm) that can be broadly categorized into three bedrock morphologies: *Flat*, *Ledge*, and *Crevice* nests (Markle et al., 2021) (see Figure 1). These nest morphologies differ from more southern turtle nesting sites in Canada in that the nest soil is quite close to the surrounding bedrock, as opposed to nests that are in the shoulders of roads, on beaches, or side embankments near water features, and is restricted to shallow deposits within the bedrock. *Flat* nest types are those where soil has accumulated on flat bedrock and typically contain the shallowest soil deposits, where there is a large amount of exposure of the nest to the open air. *Ledge* sites are those where soil deposits have accumulated up against an “L” shaped bedrock formation. In addition to the bedrock being below the soil, it is also adjacent to the nest as well, with one side more exposed to the open air. Finally, the *Crevice* sites are the most sheltered from the open air, where soil deposits have formed in deeper depressions or cracks within the bedrock, and are surrounded by bedrock on all sides, except the surface of the nest.

A total of 12 turtle nests were monitored during the 2018 and 2019 nesting season, with six nests monitored in each year. In 2018, *Emydoidea blandingii* (*n* = 2), *Clemmys guttata* (*n* = 1), and *Chrysemys picta marginata* (*n* = 4) nests were monitored. In 2019, *Chrysemys picta marginata* (*n* = 5) and *Emydoidea blandingii* (*n* = 1) nests were monitored. The 12 nests were classified as being laid in either *Flat* (*n* = 4), *Crevice* (*n* = 3), or *Ledge* (*n* = 5) sites. We recorded the number of eggs in each nest, depth to the top and bottom of the nest cavity, and depth to bedrock. At each nest, environmental variables were measured from May to September for each year including soil moisture and soil temperature at the top and bottom of the nest cavity. Temperature (°C) (iBwetland iButton, Alpha Mach, Sainte‐Julie, QC) was measured hourly and then interpolated to 15‐min intervals from sensors installed at depths that corresponded with the top and bottom of each nest. Soil moisture (5 cm probes installed horizontally at the same depths as the temperature sensors, ECH2O EC‐5 METER Environment, Pullman, WA, recorded with HOBO USB Micro Station, ONSET, Bourne, MA) was measured every 15 min. Soil moisture probes were calibrated and temperature corrected. Soil texture at each site was determined to be primarily sandy loam. See Markle et al. (2021) for more detailed information on surveying and instrumenting nest sites and the methods for egg morphological measurements.

### 2.2 Research design and conceptual model overview

Conceptual models are excellent tools to aid in understanding the different processes that are occurring within natural systems. To explain and justify our methodology, we begin with a conceptual model to outline the current understanding of how energy moves through turtle nests.

**FIGURE 2:**
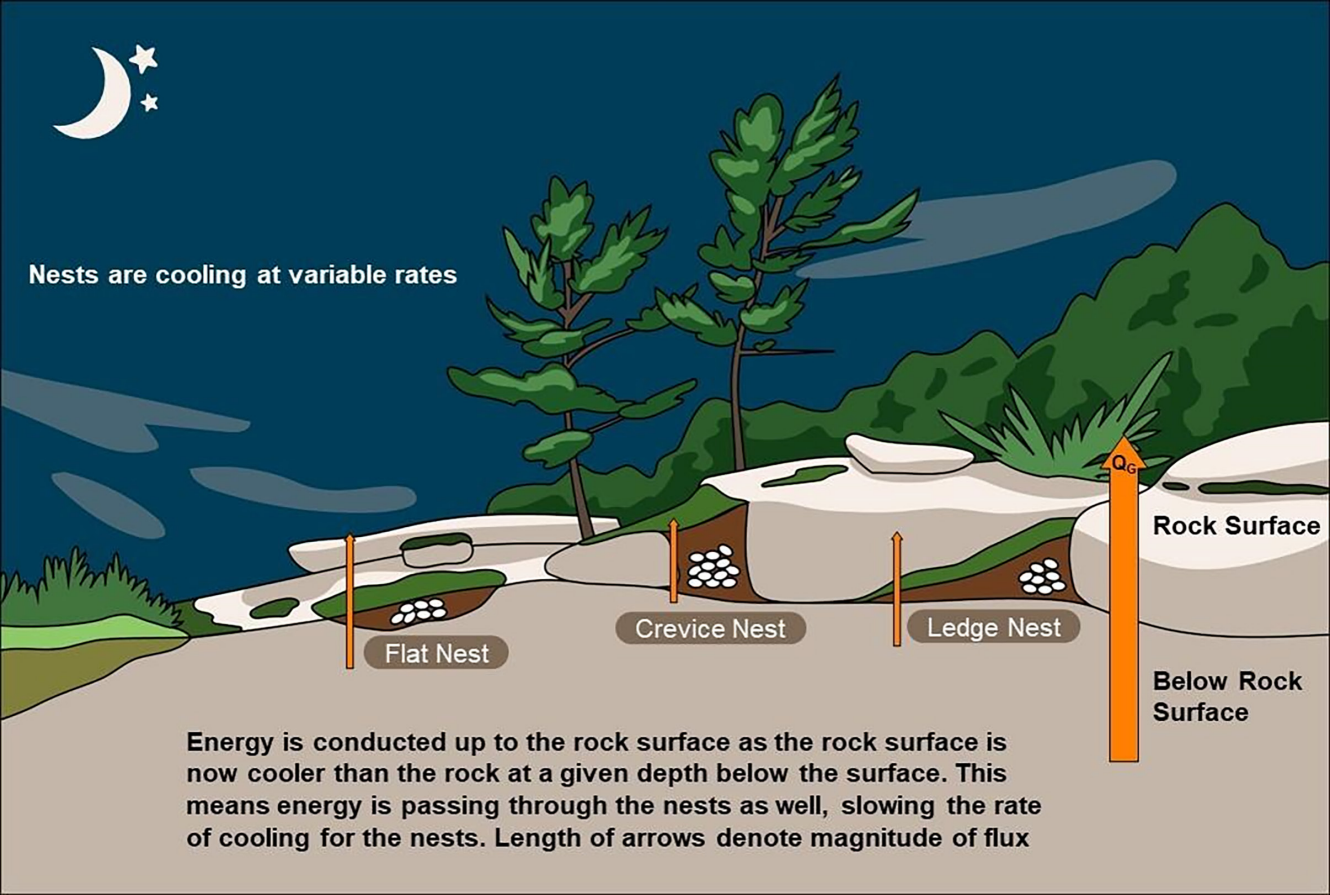
A flux reversal occurs in the evening due to a switch in temperature gradients. Energy is conducted up to the rock surface as the rock surface is now cooler than the rock at a given depth below the surface. This means energy is passing through the nests as well, slowing the rate of cooling for the nests. Length of arrows denotes magnitude of flux.

During the day, a turtle nest receives energy primarily from incoming short and long wave radiation from the sun (Figure 1). This energy is absorbed in the soil surface of the nest, where the amount absorbed is dependent on the albedo of the nest surface and the net turbulent and radiative fluxes of energy. Nesting sites with higher vegetation coverage and canopy cover will have more radiative energy intercepted (Oke, 1987). Therefore, less energy will be absorbed at a more vegetated or treed nest site, compared to a more open nest site. While there is considerable variation in vegetation cover of selected nest sites between turtle species, some turtles tend to prefer nesting in areas with more open canopy, while others will nest in sites with slightly higher canopy cover (e.g., Hughes & Brooks, 2006; Litzgus & Brooks, 2000; Markle et al., 2021; Riley & Litzgus, 2014). Vegetation cover can also vary across nest sites within a single turtle species, as vegetation structure can also impact turtle mobility and subsequently site selection (e.g., Refsnider et al., 2013). Once absorbed, the energy is conducted down through the nest cavity at a rate proportional to the temperature gradient between the top and bottom of the nest, and the nest thermal conductivity. Not all the energy will make it through to the bottom of the nest cavity. Some energy is absorbed by the soil and the eggs themselves, and is converted into sensible heat, raising the nest temperature. The more the nest temperature increases, the more energy is being absorbed by the nest itself (i.e., higher Δ*S*). The total energy (MJ t^−1^) interacting with the nest can be modeled using equation 1.

(1) $$\text{Nest Energy}=\left(\left[Q_{G}\right]+\frac{\left[\Delta S\right]}{900}\right)\times 9\times 10^{-4}$$

where *Q*_G_ (W m^−2^) is the absolute ground heat flux and Δ*S* (W m^−2^) is the absolute heat storage, 900 is the conversion factor from seconds to minutes, where 900 s are in the 15 min measurement interval, and 9 × 10^−4^ combines the time conversion factor (900) with the conversion of Joules to Megajoules (1 × 10^6^). The *Q*_G_ represents the net amount of energy that is being transferred to or from the landscape surface, therefore different nest morphologies may lead to drastically different energy regimes, in part because of potential energy contributions from the surrounding bedrock. Granite has a high thermal conductivity (≈2.75 W m^−1^ K^−1^) and a low specific heat (≈0.776 J g^−1^ cm^−3^) which means the bedrock surface can heat up very quickly leading to steep temperature gradients, conducting large fluxes during the day (Figures 1 and 2). This energy is then converted to a subsurface sensible heat, and the internal temperature of the bedrock begins to rise. This can lead to a reversal of temperature gradients in the evening at or near the surface including near the soil deposits that are being used as turtle nests (Figure 2).

Overnight, the energy that was conducted into the bedrock during the day can be conducted back up to the surface through the nests, potentially reducing nighttime temperature variability for the nests (Figure 2). Additional heat loss can occur due to longwave radiative transfer from the nests to the overlying air. These potential shifts in energy direction and absorption can be quantified using *Q*_G_ and *∆S*, which is the energy gained or lost, associated with temperature change (J cm^−3^ K^−1^). The nests will absorb a portion of the energy being conducted through and convert that energy to temperature changes.

### 2.3 Ground heat flux and heat storage

To calculate Nest Energy in Equation 1, both *Q*_G_ and *∆S* were calculated using the calorimetric method (Halliwell & Rouse, 1987; Van Huizen & Petrone, 2020) according to Equation 2:

(2) $$Q_{G}=-K_{\text{soil}}\frac{T_{z1}-T_{z2}}{z1-z2}$$

where *K*_soil_ is the thermal conductivity of the soil (J m^−1^ K^−1^) which is calculated using Equation 3:

(3) $$K_{\text{soil}}=K_{n}^{n\%}\times K_{n}^{n\%}\times K_{n}^{n\%}$$

where *K*_n_ is the thermal conductivity of each nest material (i.e., mineral soil, air, and water) and n% refers to the volumetric proportion of each material. *T*_z1_ and *T*_z2_ are the temperatures measured at the upper (Z1) and lower depth (Z2) (cm). In our case, the upper and lower depths represent the depths equivalent to the top and bottom of the nest chamber, respectively. *∆S* was then calculated using the specific heat capacity and the volume of the nest as shown below in Equations 4 and 5.

(4) $$∆S=\mathrm{VHC}\times \frac{∆T}{∆t}$$

where ∆*T*/∆*t* is the change in temperature over the specified time frame, and VHC is the volumetric heat capacity (J cm^−3^ K^−1^) calculated as shown in Equation 5:

(5) $$\;\mathrm{VHC}=\left[\frac{\left(C_{n}\times n\%\right)+\left(C_{n}\times n\%\right)+\left(C_{n}\times n\%\right)}{N}\right]\times \text{Nest Volume}$$

where *C*_*n*_ is the specific heat capacity (J g^−1^ K^−1^) of each soil constituent and *N* is the total number of soil constituents.

The observations for temperature and soil moisture were averaged for each day and then the calorimetric method was used to determine the *Q*_G_ and the *∆S* for each nest, and then averaged by nest type (i.e., ledge, crevice, flat), converted to MJ day^−1^ and plotted against the day of year to allow for the amalgamation of data across both the 2018 and 2019 seasons. In addition to the daily average, temperature and soil moisture were averaged for each hour of day across the entire time series. The calorimetric method was used to quantify average changes in *Q*_G_ and *∆S* over an average day and night.

### 2.4 Statistical analysis

We modeled changes in mean nest temperature and mean soil moisture during the incubation period using generalized linear mixed effect models (GLMM) using the glmer() function from the lme4 package (v 1.1.35.1, Bates et al., 2015) in R 4.2.3 (R Core Team, 2023). We used Gaussian distribution (identity link) to model average nest temperature and average soil moisture, respectively. Nest type (crevice, ledge, flat) was included as a fixed effect, and nest ID was included as a random effect to account for repeated measures. Turtle species were evaluated for inclusion as a random effect but found to have no variability among species and therefore not included in the final model (Figure S1).

We modeled changes in variability of ground heat flux (daily standard deviation of *Q*_G_) and heat storage (daily standard deviation of *∆S*) for each nest type (flat, crevice, ledge) throughout the incubation season using generalized additive models (GAM) using the gam() function from the mgcv package (v 1.9.1, Wood, 2011). In both models, nest type was fitted as a parametric fixed effect and day of year was fitted as a smoothing function by nest type (i.e., an interaction) to allow ground heat flux and heat storage to vary nonlinearly over time for crevice, ledge, and flat nests. Turtle species and nest ID were included as simple random smooths to account for repeated measures from the same nest site. Significance was accepted at *p*‐value <.05. All calculated average values reported below are accompanied by the standard deviation.

## 3 RESULTS

**FIGURE 3:**
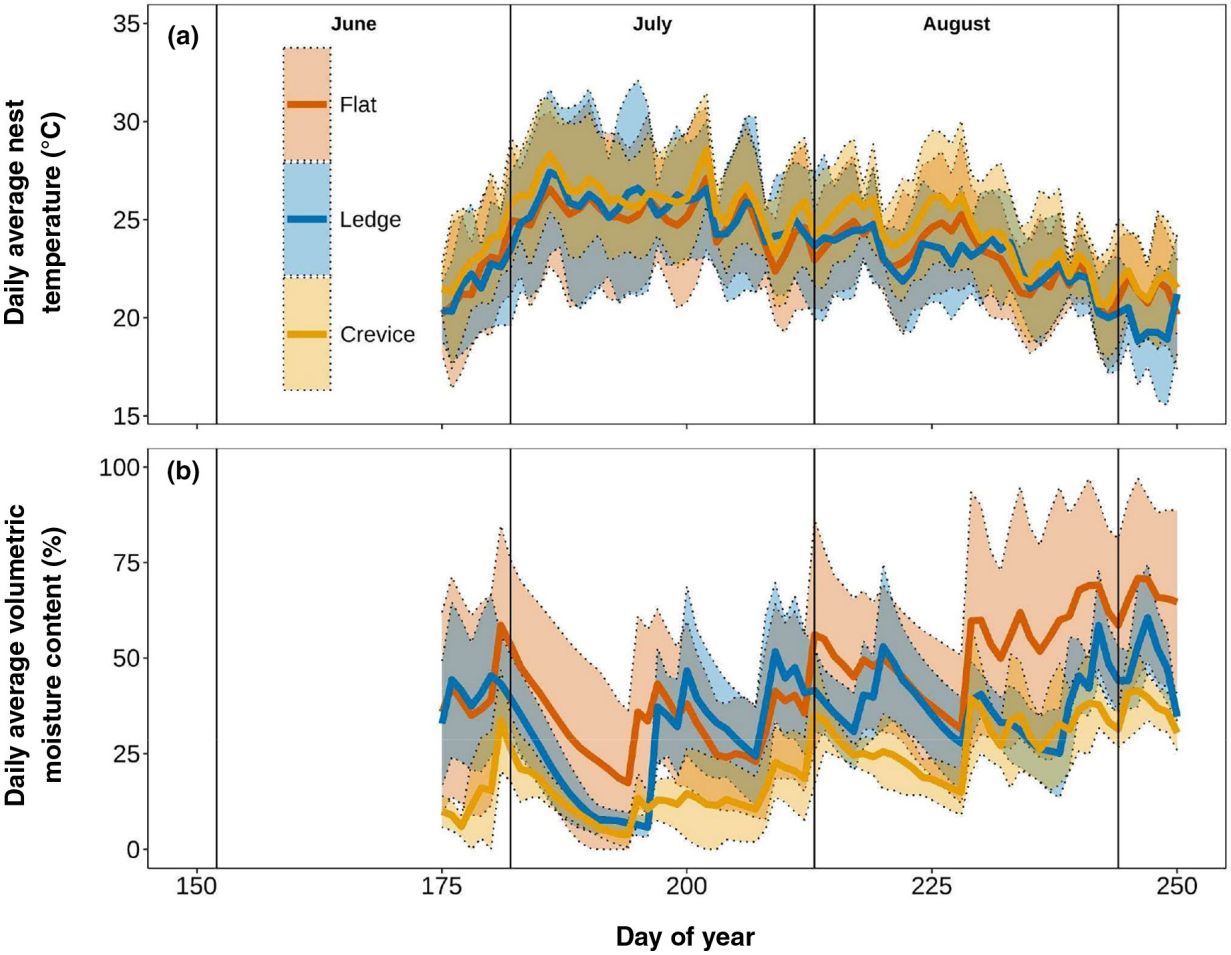
(a) Daily average nest temperature (°C) for each nest type. Each line represents an average of all upper and lower measurements taken from each individual nest for each nest type across 2018 and 2019. (b) Daily Average volumetric water content (%) represents an average of all upper and lower measurements taken from each individual nest for each nest type across 2018 and 2019. Values are calculated across two different years, and so it is only representative of general soil moisture trends to each nest type.

According to the GLMM, the *Crevice* nest morphology had a significant impact on nest temperature (est. ± SE = 1.0 ± 0.34, *t* = 3; Table S1) along with the *Flat* (est. = 23.6 ± 0.22, *t* = 107; Table S1) while *Ledge* nests did not (est. = 0.0048 ± 0.30, *t* = 0.016; Table S1). *Crevice* nest sites had an average temperature of 25.0°C (±2.2°C) which was 1.4°C warmer, on average, compared to (Figure 3a) to both *Flat* (23.6 ± 2.1°C), and *Ledge* nest sites (23.6, ±2.3°C).

Similar to nest temperature, *Crevice* and *Flat* nests exhibited the largest significant influence over soil moisture (*Crevice* est. = 1.9 ± 0.80, *t* = 2.4; *Flat* est. = 2.78 ± 0.53, *t* = 5.3; Table S2) while *Ledge* nest types showed no significant influence (est. = 0.41 ± 0.0.71, *t* = 0.58; Table S2). *Crevice* nests experienced the lowest average soil moisture (22, ±13%) compared to *Ledge* (34, ±17%) and *Flat* sites (44, ±27%), where *Flat* sites were double the moisture content on average (Figure 3b). Temperature and soil moisture were also linearly related (*R* ^2^ = .38, *p*‐value <.05) where an increase of soil moisture by 1% corresponded to a decrease in temperature of 0.08°C, meaning a higher volumetric moisture content coincided with lower temperatures across all three nest types. This is demonstrated further in the average proportions of air, water, and soil for each nest type (Table 1). *Flat* and *Ledge* nests had higher VMC and lower temperatures as well.

**TABLE 1:** Average proportions and standard deviation of nest materials that influence thermal dynamics.

Nest type	Avg soil Pct (SD)	Avg air Pct (SD)	Avg VMC Pct (SD)	Avg porosity (SD)	Avg bulk density (SD)
Crevice	0.35 (±0.05)	0.51 (±0.10)	0.22 (±0.14)	0.65 (±0.05)	904 (±182)
Flat	0.48 (±0.14)	0.29 (±0.20)	0.49 (±0.28)	0.52 (±0.14)	1247 (±525)
Ledge	0.37 (±0.03)	0.41 (±0.12)	0.36 (±0.18)	0.63 (±0.0.3)	1005 (±232)

**FIGURE 4:**
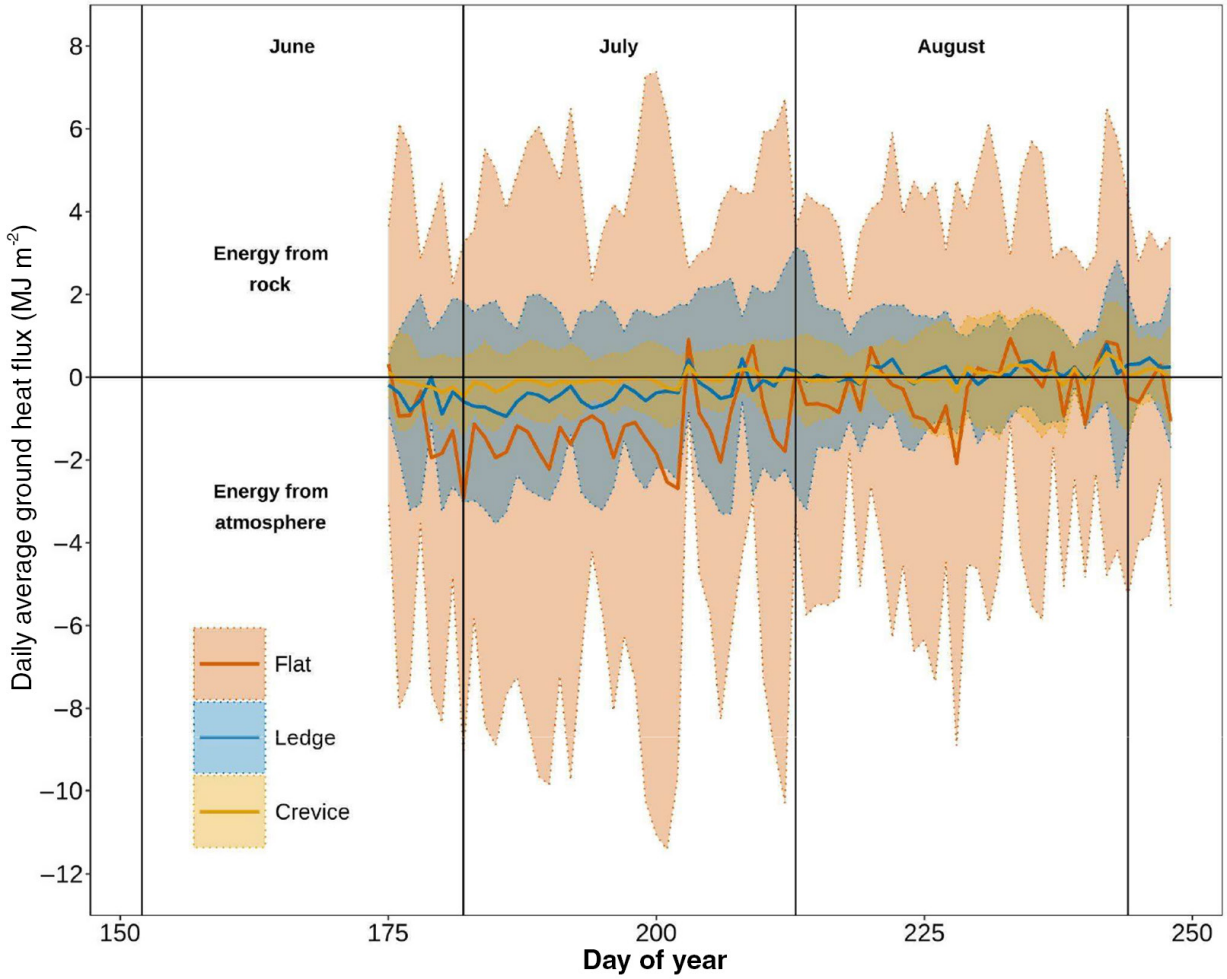
Daily Average Cumulative *Q*_G_ (MJ m^−2^) by nest type for 2018 and 2019. Shaded areas represent one standard deviation from the mean.

All three nest types appeared to have energy being directed primarily from the surface (as indicated by the negative *Q*_G_ values; Figure 4) but the *Flat* nest type saw much larger flux values (Range = 3.30 × 10^−2^ MJ—2.93 × 10^0^ MJ, average = 9.94 × 10^−1^ MJ), while the *Ledge* (Range = 7.30 × 10^−5^ MJ—9.45 × 10^−1^ MJ, average = 3.31 × 10^−1^ MJ) and *Crevice* (Range = 3.74 × 10^−3^ MJ—5.90 × 10^−1^ MJ, average = 1.56 × 10^−1^ MJ) mean nest types were much more subdued, with lower variability. Earlier in summer, between approximately DOY 175 and 200, the average fluxes were negative. However, later in the season, there is a slight increase, with more days exhibiting positive fluxes for all three nest types, indicating a potential larger influence of the rocks on *Q*_G_ as the summer season progresses. *Flat* nests experienced the highest seasonal variability (Standard Deviation ±0.68 MJ m^−2^) in *Q*_G_ (Figure 4). The variability for the *Ledge* (Standard Deviation ±0.25 MJ m^−2^) and *Crevice* (Standard Deviation ±0.12 MJ m^−2^) were comparatively lower than the *Flat* nest type. Despite differences in standard deviation, there were no significant linear effects of the nest morphology on the *Q*_G_ variability. However, when accounting for day of the year, the GAM shows that all three nest morphologies had significant nonlinear effects, modified by day of year on *Q*_G_ variability (see Table S3).

**FIGURE 5:**
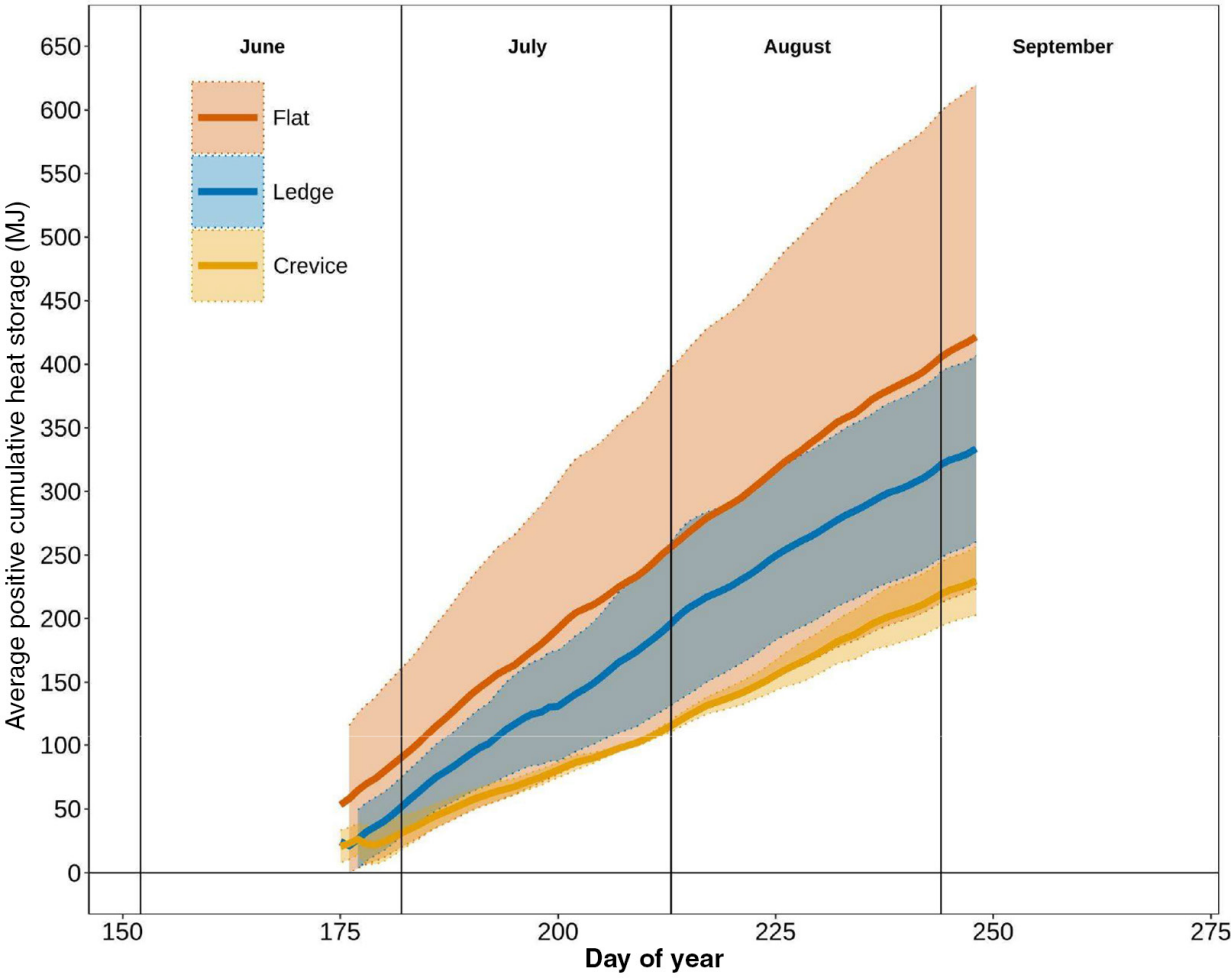
Cumulative positive (heat gained) heat storage for each nest type. Flat Nests were the highest, followed by Ledge and Crevice nests. Each line represents an average of multiple nests, and so any apparent decline is due to averaging across nests that had different incubation periods and hatching times. Shaded areas correspond to one standard deviation from the mean.

The cumulative heat storage in the *Flat* nest types (Max Value = 440 MJ) was twice as large compared to the Crevice nest type, which had the lowest maximum value (230 MJ) (Figure 5). Like daily average nest temperatures (Figure 3) and daily average *Q*_G_ (Figure 4), the magnitude of heat storage is largest in the *Flat* nest types, followed by the *Ledge* and then *Crevice* nest types.

**FIGURE 6:**
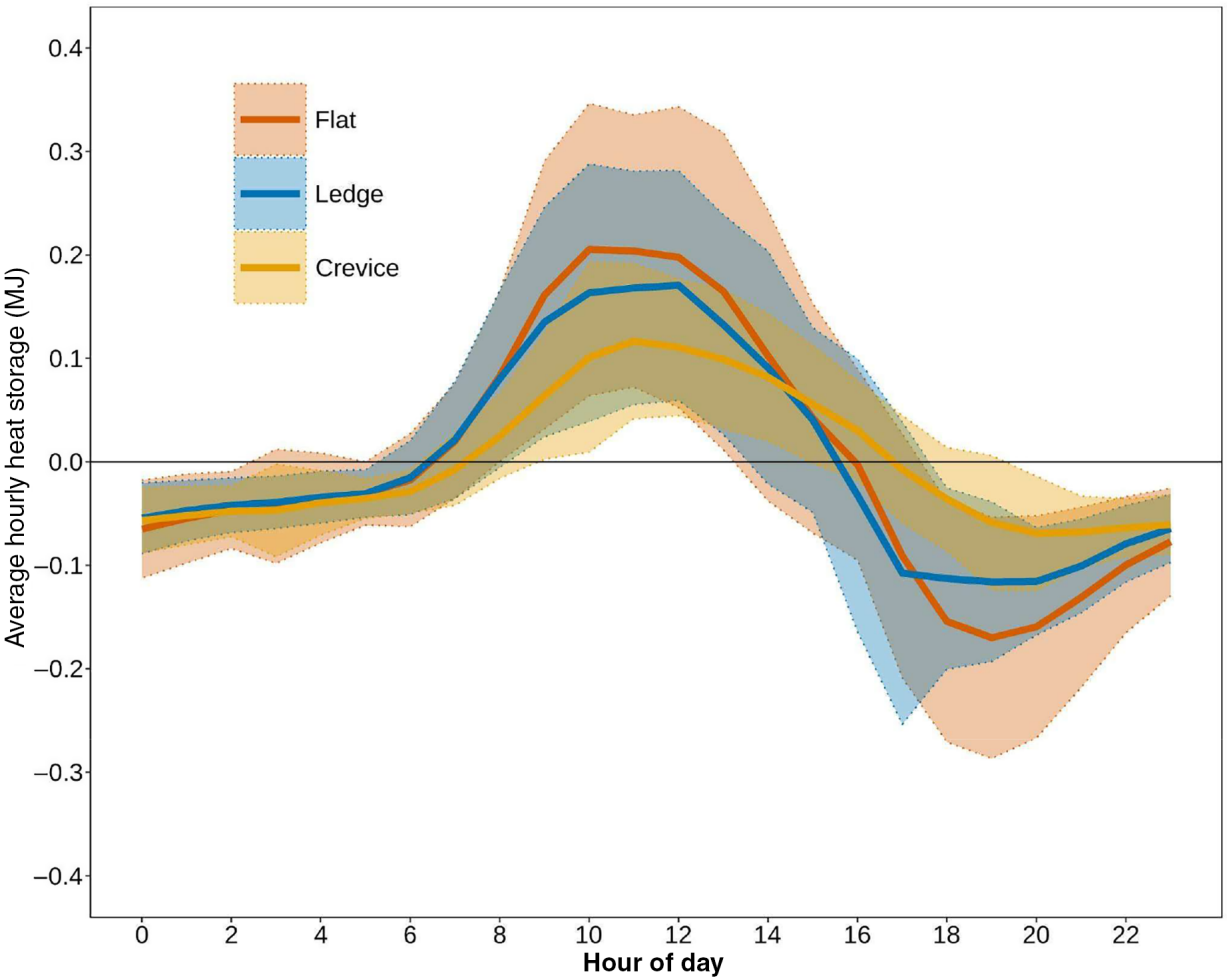
Average hourly ∆S (MJ) based on hour of day for all three nest types in 2018 and 2019. Shaded areas represent one standard deviation from the mean.

On a sub‐daily time scale, similar patterns are observed for ∆S where the largest daily heat storage gains occur in the *Flat* nests, followed by the *Ledge* and *Crevice* nests (Figure 6). However, these large gains in the Flat nests (Max = 0.21 MJ) are balanced over a 24‐h period by large ∆S losses (Min = −0.17 MJ; Figure 6). This is contrasted by the Crevice nests where the maximum value was 0.12 MJ, almost double the minimum value (−0.07 MJ), indicating a larger net gain of energy over a 24‐h period. Furthermore, these energy losses occur primarily at night for all three nest types which creates key transition periods between daytime and nighttime hour energy storage dynamics. All three nest types experience similar heat storage patterns shortly after midnight, but the slope of the lines varies across nest types as the heat storage begins to increase (Figure 6). An inflection point is reached at 6 am, which is on average, when the sun rises at this latitude. However, while the *Flat* (≈3 MJ/6 h) and *Ledge* (≈2 MJ/6 h) heat storage increase rather quickly in the morning hours (6 AM–12 PM), the *Crevice* lags (≈1.5 MJ/6 h). This lag persists into the early evening hours, where the heat storage loss is quite drastic for both the *Flat* and *Ledge* nest types, but less so for the *Crevice* nest type. These trends are further emphasized in the GAM results, where there is a significant nonlinear effect of time of day, moderated by nest morphology (see Table S4).

**FIGURE 7:**
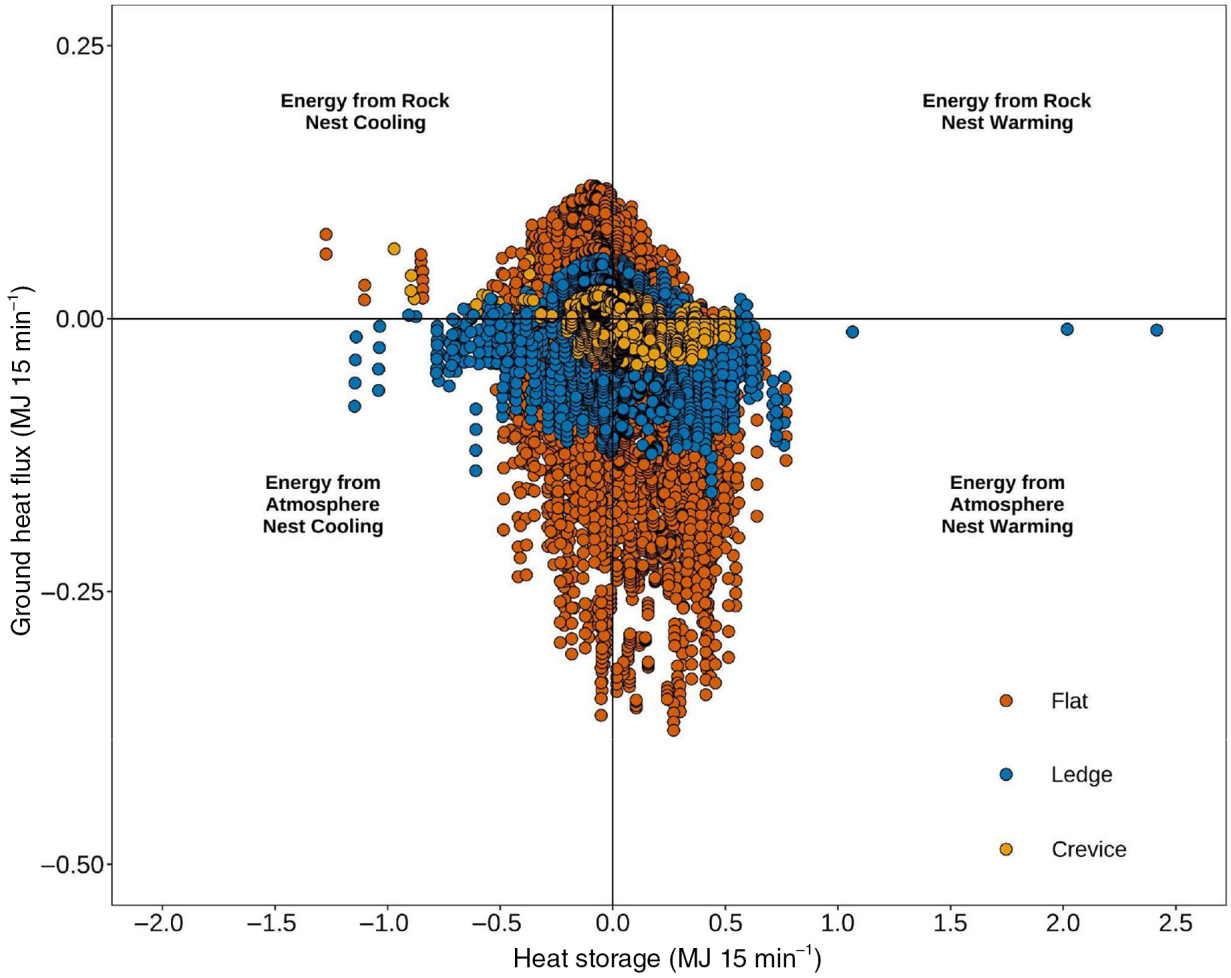
Nest Heat Storage (Δ*S*) versus Ground Heat Flux (*Q*_G_) at 15‐min intervals. Most nest warming coincides with energy being directed from the atmosphere. Table 2 shows though much of the time, energy passes through the turtle nest from the rock, with the most being the *Crevice*, *Ledge*, and then *Flat* types.

From Figure 6, it is evident that most of the time that the nest is warming (i.e., +*∆S*), and from Figure 7 that much of the energy is coming from the atmosphere (i.e., −*Q*_G_). This is contrasted by an analysis of the flux direction shown in Table 2, where for most of the time the energy is coming from the rock (average 58% of the time), however in much smaller fluxes (see Table 2).

**TABLE 2:** Percentage breakdown of energy source over the incubation period.

Nest type	Rock (%)	Atmosphere (%)	Average flux from rock (MJ/15 min)	Average flux from atmosphere (MJ/15 min)
Crevice	59	41	6.54 × 10^−3^	−9.69 × 10^−3^
Ledge	58	42	2.47 × 10^−2^	−5.03 × 10^−2^
Flat	57	43	1.09 × 10^−2^	−1.82 × 10^−2^

*Note*: Most of the time, energy is coming from the rock, albeit at a smaller flux compared to when the energy is coming from the atmosphere.

Nest fluxes at Flat nests at 15‐min intervals still had the highest magnitude, followed by the *Ledge* and finally the *Crevice* nest types (Figure 7). Furthermore, although small differences, the energy coming from the rock in the *Crevice* nest occurred 2% more often compared to the *Flat* nest and *Ledge* nest types, which also follows the pattern of proximity to the rock in the different nest types (see Figure 1).

## 4 DISCUSSION

### 4.1 Importance of nest morphology to turtle nest energy flow

The rock outcrops within this landscape appear to act as an additional heat source for the turtle nests, where rock morphology plays a key role. Not only did *Crevice* nest sites have significantly warmer nest temperatures, but they also provided the most stable energy conditions, compared to the flashier *Q*_G_ and *∆S* found in the *Flat* nest sites. *Ledge* nest sites unsurprisingly act as an intermediate nest type from a ground heat flux and heat storage perspective. These results are in line with previous work which suggests that bedrock may be an important temperature source for turtle nests at the northern limit (Bobyn & Brooks, 1994; Litzgus & Brooks, 1998) and more specifically crevice sites (Markle et al., 2021) in providing a thermally stable environment. More work is needed to quantify the thermal properties of these nests, specifically the interplay between metabolic heating and egg clutch size on the volumetric heat capacity of the nests themselves, and importance of soil moisture to thermal heating.

The *Flat* nests experienced the highest cumulative heat storage change (≈1000 MJ) yet were still slightly cooler than the *Crevice* and *Ledge* nests. This is likely due to the diurnal patterns in heat storage (Figure 6), where *Crevice* nests on average heated up and cooled down at a slower rate compared to *Flat* and *Ledge nests*. The morphology of the *Flat* nests likely contributed to less energy input from the surrounding rock overnight (when most of the energy was conducted from the rock to the nest, Figure 7), whereas the *Crevice* and *Ledge* nests had slightly more bedrock input (Table 1). Another factor could be the depth of the nests and soil moisture levels. The *Flat* nests were relatively shallow (7.5 cm, ±1.7 cm) compared to the *Crevice* (17.9 cm, ±2.2 cm) and *Ledge* nests (16.6 cm, ±3.5 cm). These shallower nests will heat up and cool down quicker, just by virtue of having a smaller nest volume (Table S5), whereas the *Crevice* nests, which are the deepest, were the slowest to change (Figure 6).

Interestingly, soil moisture was not enough of a buffer in the *Flat* nests to slow temperature changes. Despite having the highest relative soil moisture of the three nest types (Figure 2a), it had the flashiest *Q*_G_ (Figure 3). Typically, an increase in soil moisture buffers against rapid temperature changes (e.g., Van Huizen et al., 2021). However, it could be because the absolute amount of water was lower in the *Flat* nests, diminishing any thermal regulation capacity. The *Flat* nests also had the highest average thermal diffusivity (6.2 × 10^−1^ ± 1.4 × 10^−1^ m^2^ s^−1^) compared to the *Crevice* (=5.2 × 10^−7^ ± 5.5 × 10^−7^ m^2^ s^−1^) and *Ledge* (5.7 × 10^−7^ ± 6.8 × 10^−7^ m^2^ s^−1^) nests, which means that the entire *Flat* nest can experience temperature changes more rapidly (Oke, 1987). For the *Crevice* nest, where the thermal diffusivity was lower, it is possible the lower depth temperature measurements were not as impacted by energy coming from the surface due to the lower thermal diffusivity. Since both the upper and lower temperature values were averaged for the *Q*_G_ and Δ*S* calculations, this could lead to more moderate changes in temperature (Figure 6).

It should be noted that although a greater proportion of time is spent when the energy is coming from the direction of the rock, the flux is still comparatively smaller then when it is coming from the atmosphere (see Table 2). This is because energy is typically being conducted into the nests from the rock at night (Figure 7), when temperature gradients are less steep leading to less conducted energy overall. Most of the energy has been used/transformed throughout the daytime hours. Despite these smaller amounts of energy, it is likely that they are contributing to the slower changes in heat storage for the *Crevice* nest (Figure 6) as other nest thermal properties that affect the heat storage capacity are relatively static overnight (i.e., soil moisture).

While our study focuses on heat conduction through the rock, it is also possible that there is additional heating occurring via thermal conduction in the air as well, albeit in quite small amounts. Another potential confounding factor is the metabolic energy of the developing turtle embryos within the nests. While the metabolic heating of the nest via the embryo has been theorized to occur (e.g., Godfrey et al., 1997), it is only recently that studies have been able to quantify it. A study by Massey et al. (2019) has shown that there can be an increase in nest temperature by 0.3°C. While that study was focused on snapping turtles, their results provide insight into this process. For our study, though, we attempted to mitigate these effects by placing the temperature probes next to the cavity as opposed to inside the nest cavity. This may result in a slight overestimation of energy fluxes, as the soil does not account for the individual thermal properties of the eggs, which could increase the volumetric heat capacity of the soil volume. This represents a potential important area of future research to parameterize for any nest energy modeling work.

The soil type and texture within the nest itself can also impact the energy dynamics of a nest. While this study focuses on turtles nesting in a rock barren landscape, globally, turtles nest in a variety of habitats (e.g., beaches (e.g., Madden et al., 2008), roadside embankments (e.g., De Solla & Gugelyk, 2018), open fields (e.g., Linck et al., 1989), and adjacent to or in wetlands (e.g., Dupuis‐Désormeaux et al., 2019)). The methodology used here to quantify the heat flux through these turtle nests can be readily adapted to nests over a broader geographical range and with varying soil constituents. Turtle nest sites for freshwater and marine turtles are often situated in large sites with sandy soil, where there is no influence of the bedrock. These sites may undergo more variable heating conditions, similar to the Flat nests. Yet, it is also possible that other environmental factors may mitigate the larger heat fluctuations, such as shallow subsurface through‐flow of groundwater, or differing vegetation cover. For example, in areas with invasive phragmites, extensive shading and subsequent cooling are detrimental to turtle nests (Bolton & Brooks, 2010). Understanding the potential for ecosystem redundancy influencing turtle nest site selection should continue to be an area of ongoing research.

### 4.2 Implications for modeling

This study also shows a valuable connection between abiotic and biotic studies in the field of ecohydrology. By directly calculating the *Q*_G_ and Δ*S*, the impact of the surface energy budget can be directly linked to biological processes. Models such as CRHM (Pomeroy et al., 2022) and RAVEN (Craig et al., 2020) can provide direct links, by allowing the user to write their own custom processes, that are linked to existing surface energy budget calculations. Indeed, related work has already been completed (e.g., Kearney & Enriquez‐Urzelai, 2023). Previous modeling work on egg embryo development and modeling (e.g., Cagle et al., 1993) could then be integrated into an already functioning ecohydrological model.

Beyond linking with other models, more advanced 3D heat transfer modeling may be useful to further elucidate controls on hatch success, and just how much of an impact the surrounding rock may have on turtle nest success. This study captured the dominant vertical flux (i.e., energy coming from above or below the nest), and did not directly measure energy inputs from adjacent rock. However, the more moderate heat storage changes in the *Crevice* nests are likely due to the integration of this lateral movement of energy. Being able to fully capture energy inputs from all rock orientations can provide insights into potential heat transfer anisotropy within the nests and may help in further differentiating any potential differences between *Ledge* and *Crevice* nest types. Such work would also contribute to a deeper understanding of the energy transfer in rock barren ecosystems, an area that is facing increasing pressures from development (Government of Ontario, 2021) and climate change (Zhang et al., 2019).

### 4.3 Implications for conservation and scaling

Finally, it will be important to scale up the ability to identify these different nest types for conservation. Of course, this is complicated by smaller scale differences in nest elevation. There is not much distinct elevation difference between *Crevice*, *Ledge*, and *Flat* nest types, and it would require submeter LiDAR resolution, likely in conjunction with surface characteristics such as vegetation cover and type. Some of this challenge can be mitigated using remotely piloted aircraft systems, along with ground truthing of potential nest sites. In this way, key areas can be identified for conservation purposes. It may also be that such fine scale is not needed, and simply identifying rock barren habitat where reptile refugia can be found using topographic indices (i.e., prevalence of south facing slopes, thresholds for canopy cover, topographic wetness index). These parameters could be adjusted depending on the species studied and their location, to account for geographic variation within species nesting sites (Morjan, 2003). Either way, large‐scale surveys would be a benefit beyond reptile ecology, as knowledge of the nesting sites' spatial distribution could give an indication of the spatial distribution of soil depth across rock barrens, which would be integral for regional carbon storage studies, creating another collaboration link between different environmental scientific disciplines. Future studies should continue to look at developing these types of indices to enable the rapid mapping of potential conservation sites.

## 5 CONCLUSIONS

The work completed here contributes to a growing body of literature on the abiotic‐biotic feedbacks within the herpetological sciences and integrates key ecohydrological processes. Such work is important to continue as ecohydrological models are developed to include more processes, and better link biotic and abiotic processes. Furthermore, this work highlights important considerations for both the ecologist and hydrologist and represents important cross collaborations within the sciences.

The work itself highlights the continued need for research on turtle nesting behavior, which can be used in conservation efforts to protect existing natural nest sites and inform nest habitat restoration. The energy fluxes calculated here are a first step in quantifying the interlocking processes between turtle nest conditions and the surface energy budget. Future work should continue to investigate these processes so that we can understand how nest habitat suitability might change as the climate continues to warm. The additional energy inputs from the surrounding bedrock may be beneficial to turtle species in today's climate, but as the temperatures continue to warm, exposed rock will heat up at a faster rate. There may be a tipping point reached where suddenly the rock provides too much energy and warmer temperatures leading to a shift in sex ratios and hatch success. Conversely, predicted increased precipitation may lead to wetter soil conditions, buffering these thermal changes. Such nonlinear effects in nesting habitat are poorly understood and may impact the spatial distribution of suitable nesting habitat. As such they represent an important area of research that needs to be explored.

## AUTHOR CONTRIBUTIONS

**Brandon Van Huizen:** Conceptualization (lead); formal analysis (lead); investigation (lead); methodology (lead); visualization (lead); writing – original draft (lead); writing – review and editing (lead). **Chantel E. Markle:** Conceptualization (supporting); data curation (lead); formal analysis (supporting); funding acquisition (supporting); investigation (supporting); writing – original draft (supporting); writing – review and editing (supporting). **Paul A. Moore:** Formal analysis (supporting); investigation (supporting); writing – original draft (supporting); writing – review and editing (supporting). **James M. Waddington:** Funding acquisition (lead); writing – original draft (supporting); writing – review and editing (supporting).

## CONFLICT OF INTEREST STATEMENT

The authors declare that they have no known conflict of interest or personal relationships that could have appeared to influence the work reported in this paper.

## Supporting information


Data S1

## Data Availability

The data that support the findings of this study are available from the corresponding author upon reasonable request.
