# Supplementary material for: Bedrock morphology influences rock barrens turtle nesting habitat energy dynamics
Source: Ecol Evol. 2024 Apr 1;14(4):e11183. doi: 10.1002/ece3.11183 (PMC10985362; doi:10.1002/ece3.11183)
Supplement: Supplementary file 1 — Data S1 [file ECE3-14-e11183-s001.docx]

Supplementary Material

**Bedrock morphology influences rock barrens turtle nesting habitat energy dynamics**

**Brandon Van Huizen^1,2*^, Chantel E. Markle^2,3^, Paul A. Moore^1^, James M. Waddington^1^**

^1^ School of Earth, Environment & Society, McMaster University, 1280 Main Street, Hamilton, ON, Canada, L8S 4K1.

^2^ Department of Geography & Environmental Management, University of Waterloo, 200 University Ave West, Waterloo, ON, N2L 3G1, Canada

^3^ School of Environment, Resources and Sustainability, University of Waterloo, Waterloo, ON, N2L 3G1, Canada

*** Corresponding author:** Brandon Van Huizen, Email: vanhuizb@mcmaster.ca

Several different models were run to elucidate the potential influences of nest morphology on various turtle nest ecohydrological parameters. Using the glmer() function from the lme4 package (Bates et al., 2015), a Generalized Linear Mixed Effect Model (GLMM) was fit to the nest soil moisture and temperature data. General Additive Models (GAM) for *Q_G_* and *∆S* were fit using the gam() function from the mgcv package (Wood, 2011). A complete breakdown of each model is shown below in the following sections. A breakdown of average nest volumes is also included.

**Table S-1: Results from the Generalized Linear Mixed Effect Model, showing that Crevice and Flat both had a significant effect on nest temperature. Note that the intercept corresponds to the Flat nest type.**

| **Formula: Temp.nest.mean~nest.type+(1\|site.abs)** | | | | | | |
| --- | --- | --- | --- | --- | --- | --- |
| **Fixed effects:** | | | | | | |
| **Groups Name** | **Estimate** | **Std. Error** | ***t*-value** | **Significant at 95% conf when *t*-value>2** | | |
| *(Intercept)* | 23.63 | 0.22 | 107.19 | *** | | |
| *Ledge* | 0.0050 | 0.30 | 0.017 |  | | |
| *Crevice* | 1.00 | 0.34 | 2.95 | * | | |


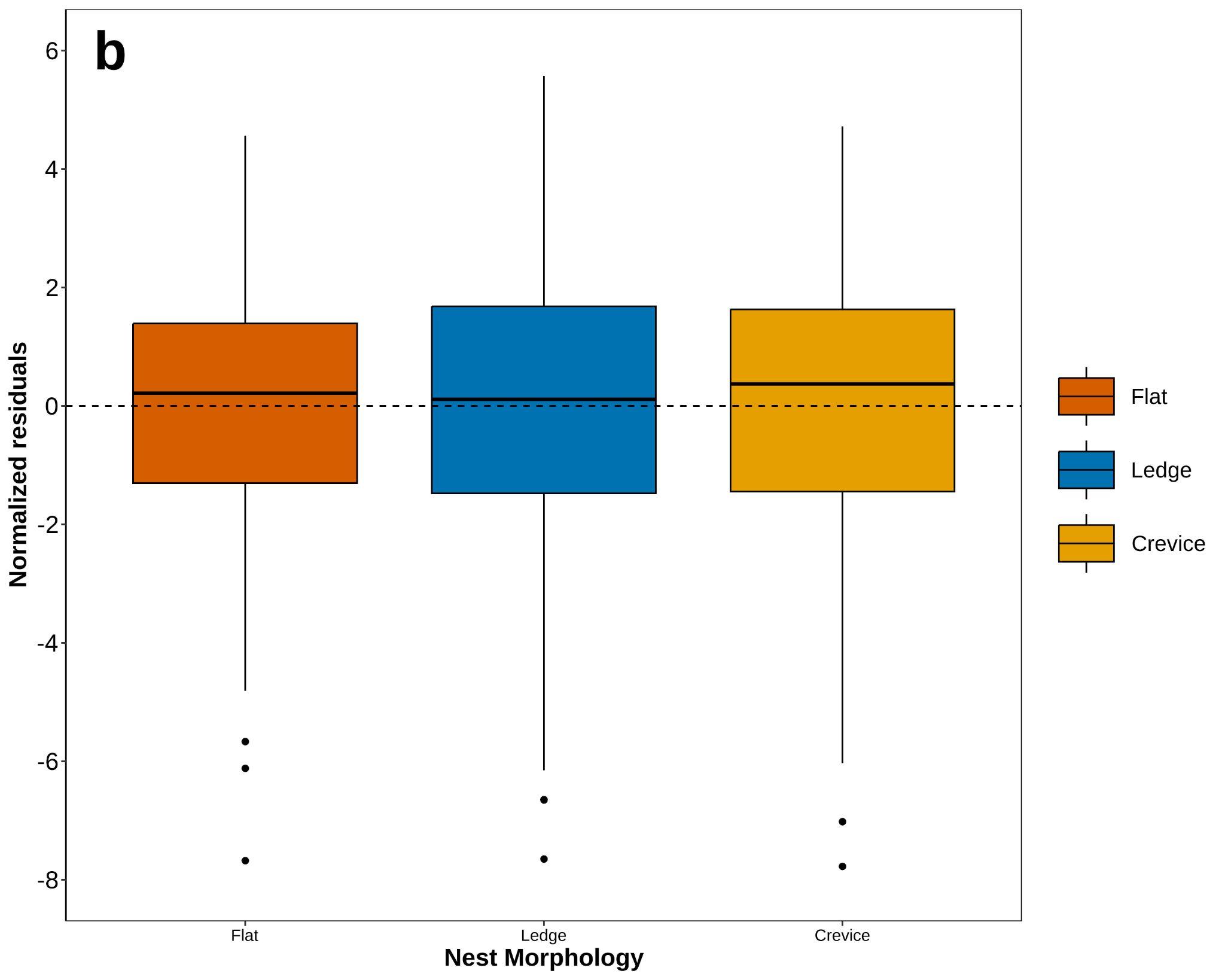

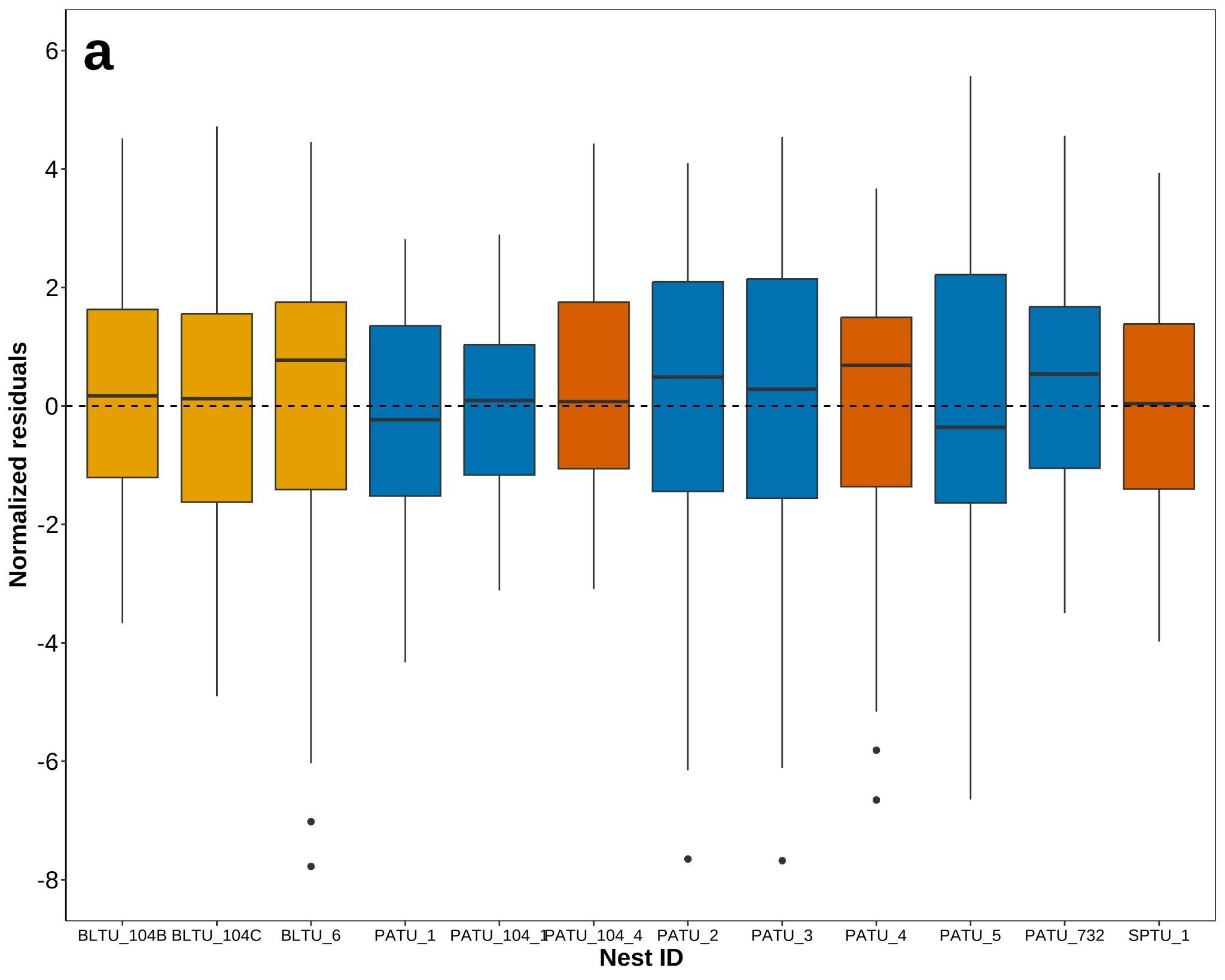


**Figure S-1: Comparison of model residuals from the GLME for both the random effect of nest site (ID) and the fixed effects of Nest morphology (panel b). Both show no obvious trends between Covariates and Nest temperature. Note that the colors in both plots correspond to nest morphology, see legend in panel b.**

**Table S-2 Results from the Generalized Linear Mixed Effect Model, showing that Crevice and Flat both had a significant influence on nest temperature. Note that the intercept corresponds to the Flat nest type.**

| **Formula: Moist.nest.mean~nest.type+(1\|site.abs)** | | | | | | |
| --- | --- | --- | --- | --- | --- | --- |
| **Fixed effects:** | | | | | | |
| **Groups Name** | **Estimate** | **Std. Error** | **t-value** | **Significant at 95% conf when t-value>2** | | |
| *(Intercept)* | 2.7811 | 0.5289 | 5.258 | *** | | |
| *Ledge* | 0.4125 | 0.7071 | 0.583 |  | | |
| *Crevice* | 1.9213 | 0.7961 | 2.413 | * | | |

**Figure S-2: Comparison of model residuals from the GLME for both the random effect of nest site (ID) and the fixed effects of Nest morphology (panel b). Both show no obvious trends between Covariates and Soil Moisture. Note that the colors in both plots correspond to nest morphology, see legend in panel b.**
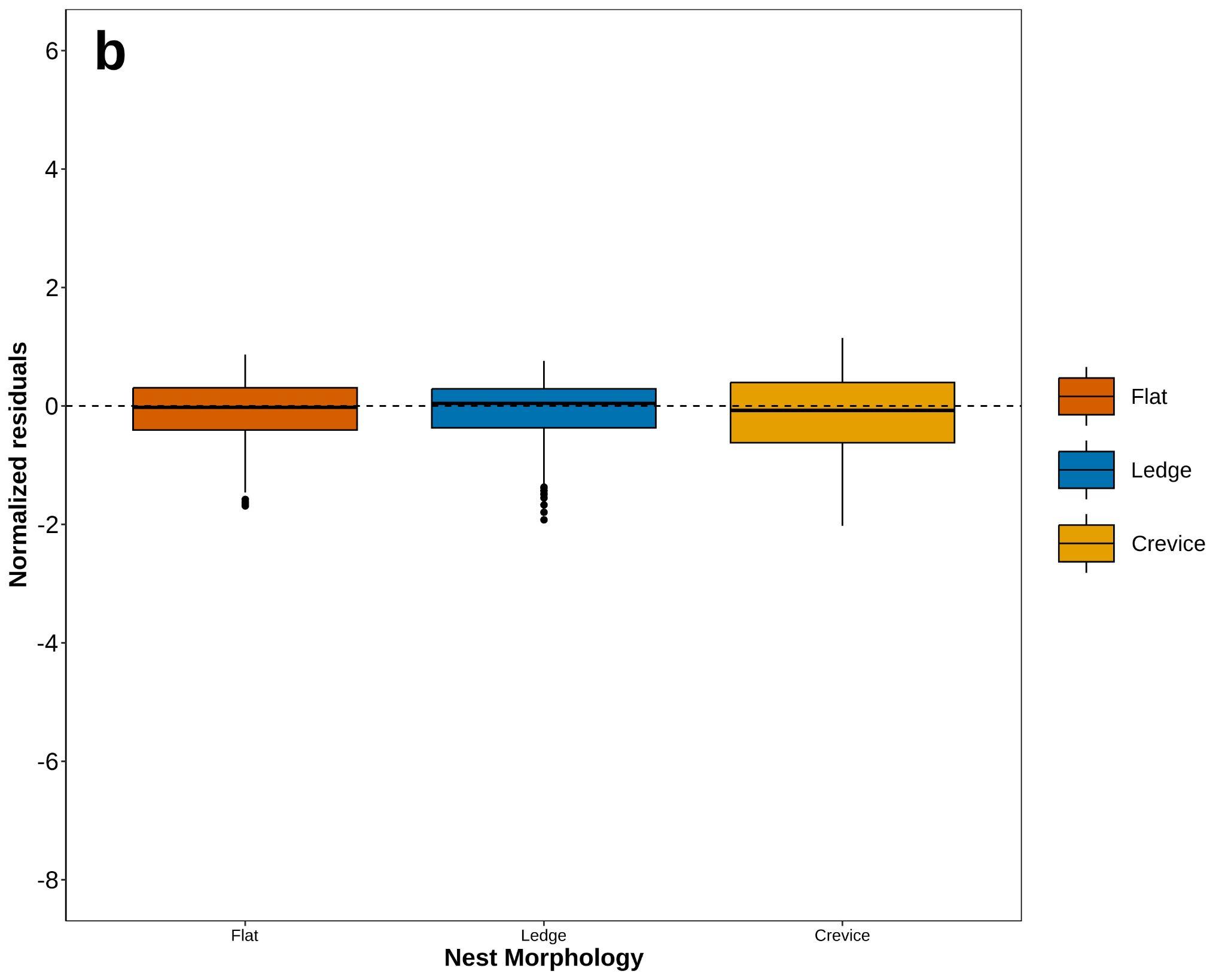

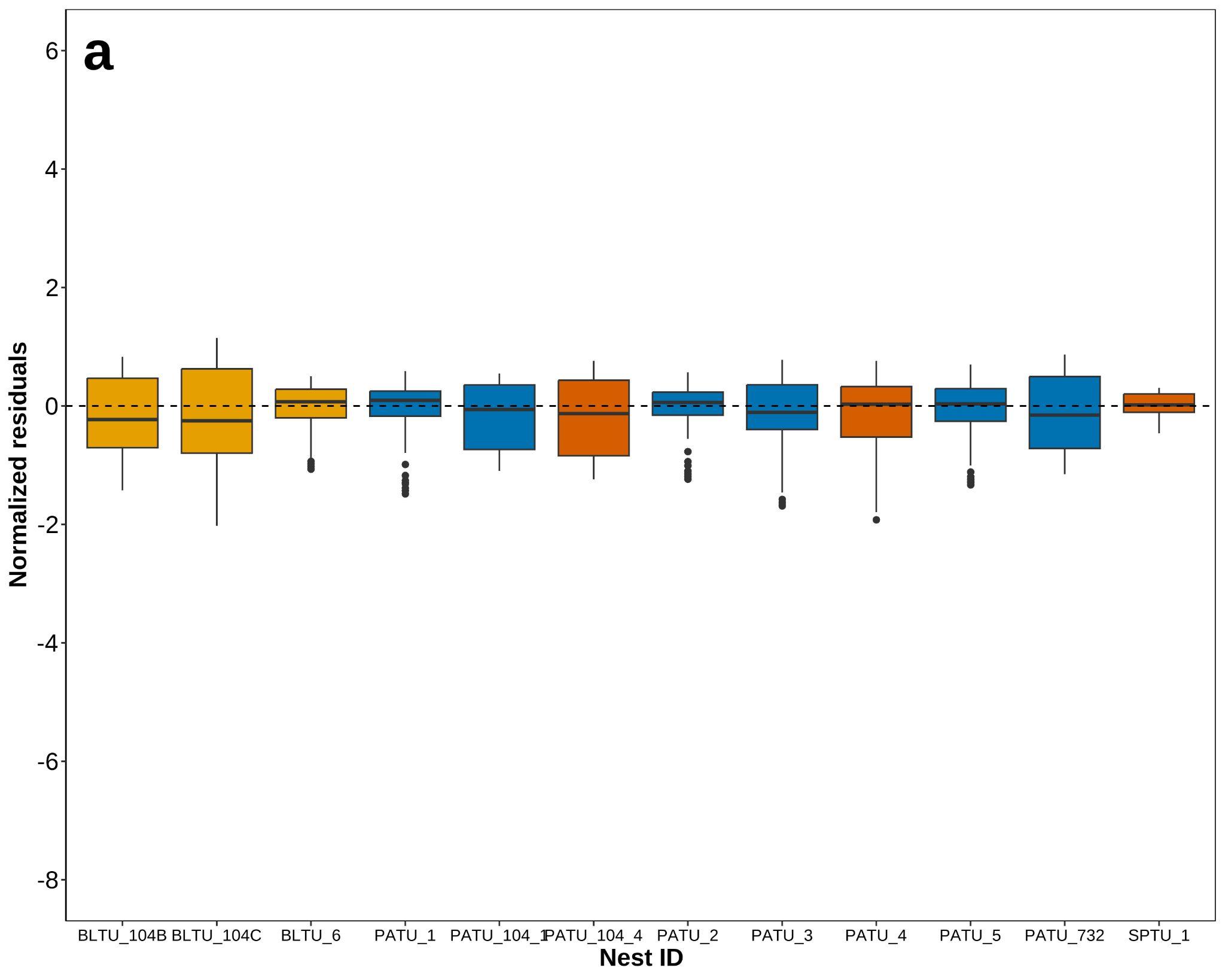


**Table S-3-1 GAM Model Results for controls on *Q_G_* Standard Deviation**

| **Approximate**  **significance of smooth terms:** | | | | | | |
| --- | --- | --- | --- | --- | --- | --- |
|  | **edf** | **Ref.df** | **F** | **p-value** | | |
| **s(DOY):nest.typeFlat** | 1.0001 | 1 | 4.282 | 0.03881 * | | |
| **s(DOY):nest.typeLedge** | 3.564 | 4.416 | 3.6 | 0.00635 ** | | |
| **s(DOY):nest.typeCrevice** | 5.2563 | 6.373 | 5.677 | 5.78E-06 *** | | |
| **s(site.abs)** | 7.9632 | 9 | 321.277 | < 2e-16 *** | | |
| **s(species)** | 0.9695 | 1 | 1777.541 | < 2e-16 *** | | |
|  | **Estimate** | **Std. Error** | **t value** | **Pr(>\|t\|)** | | |
| **(Intercept)** | 5.5144 | 3.3181 | 1.662 | 0.0969 | | |
| **nest.typeLedge** | -0.7953 | 0.6915 | -1.15 | 0.2504 | | |
| **nest.typeCrevice** | -4.6921 | 5.7149 | -0.821 | 0.4119 | | |

**Table S-3-2 GAM k estimations for model fit on *Q_G_* Standard Deviation**

| **Basis dimension (k)**  **checking results. Lowp-value (kindex<1) may indicate that k is too low,**  **especially if edf is close to k'** | | | | | | |
| --- | --- | --- | --- | --- | --- | --- |
|  | **k'** | **edf** | **k-index** | **p-value** | | |
| **s(DOY):nest.typeFlat** | 9 | 1 | 0.65 | <2e-16 *** | | |
| **s(DOY):nest.typeLedge** | 9 | 3.564 | 0.65 | <2e-16 *** | | |
| **s(DOY):nest.typeCrevice** | 9 | 5.256 | 0.65 | <2e-16 *** | | |
| **s(site.abs)** | 12 | 7.963 | NA | NA | | |
| **s(species)** | 3 | 0.969 | NA | NA | | |
| **R-sq. (adj):** 0.783 | | **Deviance explained=**81.9% | | | | |

While the k value check above suggests that our k-value chosen for the smoothing parameter is too low, increasing the initial k-value did not improve the k-index. This situation was deemed acceptable, following guidelines from Wood, (2017), who said that the more important metric is that the edf value is smaller than k’, which is the case with this model. The suitability of this model is further backed up by the distribution of the residuals and the modeled versus observed results shown below in Figure S-3.


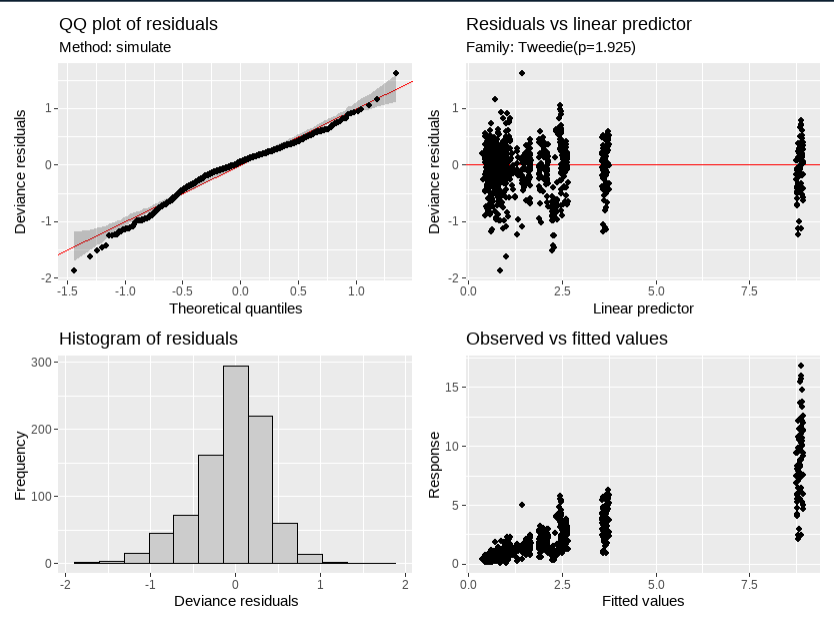


**Figure S-3 Showing the plots of the residuals being normally distributed with little trend (upper left, upper right, bottom left), and a general linear fit for the observed *Q_G_* Standard Deviation versus the modelled values (lower left).**

**Table S-4-1 GAM Model Results for controls on *∆S* Standard Deviation**

| **Approximate**  **significance of smooth terms:** | | | | | | |
| --- | --- | --- | --- | --- | --- | --- |
|  | **edf** | **Ref.df** | **F** | **p-value** | | |
| **s(DOY):nest.typeFlat** | 6.1321 | 7.224 | 16.001 | < 2e-16 *** | | |
| **s(DOY):nest.typeLedge** | 6.1095 | 7.199 | 22.032 | < 2e-16 *** | | |
| **s(DOY):nest.typeCrevice** | 6.4757 | 7.585 | 8.815 | < 2e-16 *** | | |
| **s(site.abs)** | 7.0131 | 8 | 24.985 | 0.00021 *** | | |
| **s(species)** | 0.6321 | 1 | 80.937 | 0.10267 | | |
|  | **Estimate** | **Std. Error** | **t value** | **Pr(>\|t\|)** | | |
| **(Intercept)** | 69045 | 13926 | 4.958 | 1.37e-06 *** | | |
| **nest.typeLedge** | -1150 | 10922 | -0.105 | 0.916 | | |
| **nest.typeCrevice** | -27818 | 22699 | -1.226 | 0.222 | | |

**Table S-4-2 GAM Model Results for controls on *∆S* Standard Deviation**

| **Basis dimension (k)**  **checking results. Lowp-value (kindex<1) may indicate that k is too low,**  **especially if edf is close to k'** | | | | | | |
| --- | --- | --- | --- | --- | --- | --- |
|  | **k'** | **edf** | **k-index** | **p-value** | | |
| **s(DOY):nest.typeFlat** | 9 | 6.132 | 1.08 | 0.94 | | |
| **s(DOY):nest.typeLedge** | 9 | 6.109 | 1.08 | 0.88 | | |
| **s(DOY):nest.typeCrevice** | 9 | 6.476 | 1.08 | 0.92 | | |
| **s(site.abs)** | 11 | 7.013 | NA | NA | | |
| **s(species)** | 3 | 0.632 | NA | NA | | |
| **R-sq. (adj):** 0.646 | | **Deviance explained=**75.9% | | | | |


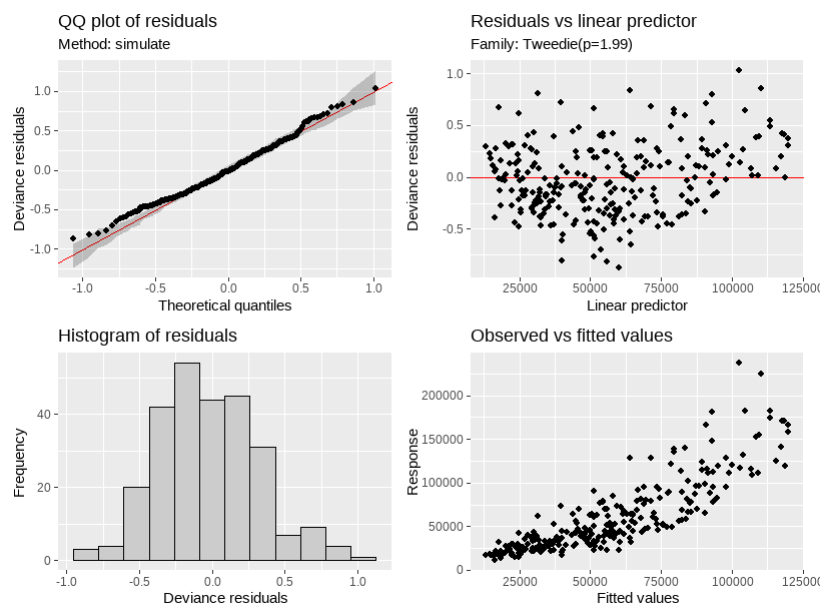


**Figure S-4 Showing the plots of the residuals being normally distributed with little trend(upper left, upper right, bottom left), and a general linear fit for the observed *∆S* Standard Deviation versus the modelled values (lower left).**

**Table S-5: Mean Nest Proportions for Blanding’s and Painted Turtles. There was only one Spotted turtle nest, and so was not included in the averages reported below.**

| **Species** | **Mean (mm) chamber width (range)** | **Mean depth (mm)to eggs (range)** | **Mean depth (mm) to bottom of nest chamber (range)** | **Mean (mm) total chamber height (range)** | **Mean depth (mm) to bedrock at nest center (range)** | **Mean (cm3) chamber volume (range)** |
| --- | --- | --- | --- | --- | --- | --- |
| **Blanding’s turtle** | 86 (80-93) | 58 (50-75) | 112 (100-135) | 53 (50-60) | 202 (205-222) | 216 (168-255) |
| **Painted turtle** | 80 (60-120) | 49 (45-55) | 92 (80-105) | 43 (35-55) | 112 (80-166) | 164  (71-339) |

**Works Cited**

Douglas Bates, Martin Maechler, Ben Bolker, Steve Walker (2015). Fitting Linear Mixed-Effects Models Using lme4. Journal of Statistical Software, 67(1), 1-48. doi:10.18637/jss.v067.i01.

Wood, S.N. (2011) Fast stable restricted maximum likelihood and marginal likelihood estimation of semiparametric generalized linear models. Journal of the Royal Statistical Society (B) 3(1):3-36

Wood, S.N. (2017) Generalized Additive Models: An Introduction with R (2nd edition). CRC/Taylor & Francis. <https://www.maths.ed.ac.uk/~swood34/>
